# Difficult Implanon Extraction Resulting in Ulnar Neuropathy: Clinical Insights

**DOI:** 10.7759/cureus.80908

**Published:** 2025-03-20

**Authors:** Chai Jia Lik, Lim Chia Hua, Fredy Arianto, Shalimar Abdullah, Jamari Sapuan

**Affiliations:** 1 Department of Orthopedics and Traumatology, Faculty of Medicine, Universiti Kebangsaan Malaysia, Kuala Lumpur, MYS; 2 Department of Orthopedics and Traumatology, Eka Hospital Bekasi, West Java, IDN

**Keywords:** contraceptive implant, implant removal, neuropathy, primary healthcare, ulnar nerve

## Abstract

Implanon NXT is a widely used contraceptive implant that is generally inserted and removed safely by primary healthcare providers. Although complications are rare, providers must remain vigilant for potential adverse events. One such complication is ulnar neuropathy, a rare but serious nerve injury that can occur during the removal process due to inadvertent nerve impingement or trauma. This condition may result in significant functional impairment and chronic pain, representing a catastrophic outcome for the patient if not managed appropriately. The procedure's success relies heavily on meticulous technique, proper training, and the ability to recognize early signs of complications. In cases where removal proves difficult or complications arise, timely referral to a specialty center is critical. Such referrals ensure that patients receive expert care, potentially mitigating long-term sequelae and optimizing overall outcomes.

## Introduction

Subdermal contraceptive implants have emerged as a popular alternative to traditional methods such as oral contraceptive pills and intrauterine devices. Implanon NXT, a radio-opaque and non-biodegradable progestagen-only implant, is preloaded in a sterile, ready-to-use disposable applicator for ease of use. It is typically inserted subdermally into the medial aspect of the non-dominant upper arm, between the biceps and triceps muscles, making the procedure both minimally invasive and accessible [1].

In Malaysia, Implanon NXT has become a favored choice among contraceptive options. Given its widespread adoption, it is crucial for healthcare providers to undergo proper training to master the techniques for both insertion and removal. Although complications such as ulnar neuropathy are rare, certain factors during the removal process can predispose patients to this serious nerve injury. Notably, there is a paucity of literature addressing nerve injuries following Implanon NXT removal in this region, underscoring the need for further research and careful clinical practice.

## Case presentation

We report a case of a 28-year-old woman who had an Implanon device inserted three years ago for contraception, with an uneventful insertion. When she later presented to the same general practitioner for removal under local anesthesia, the procedure was unsuccessful. A subsequent attempt at another center using ultrasound guidance also failed. Following these two unsuccessful removal attempts, she developed weakness in her left-hand grip and reduced sensation over the ring and little fingers, prompting referral to a tertiary center specializing in hand and microsurgery for assistance with removal.

On examination, the patient was petite and had a 0.5-cm incisional scar on the distal medial aspect of her left arm. The Implanon implant was not palpable. On hand function evaluation, the flexor digitorum profundus (FDP) strength was rated as M4 for the ring finger and M3 for the little finger, while the abductor digiti minimi (ADM) muscles had no measurable power. Sensory testing demonstrated diminished sensation over the ulnar nerve distribution and its dorsal branch, accompanied by a positive Froment sign, findings suggestive of a high lesion ulnar neuropathy.

**Figure 1:**
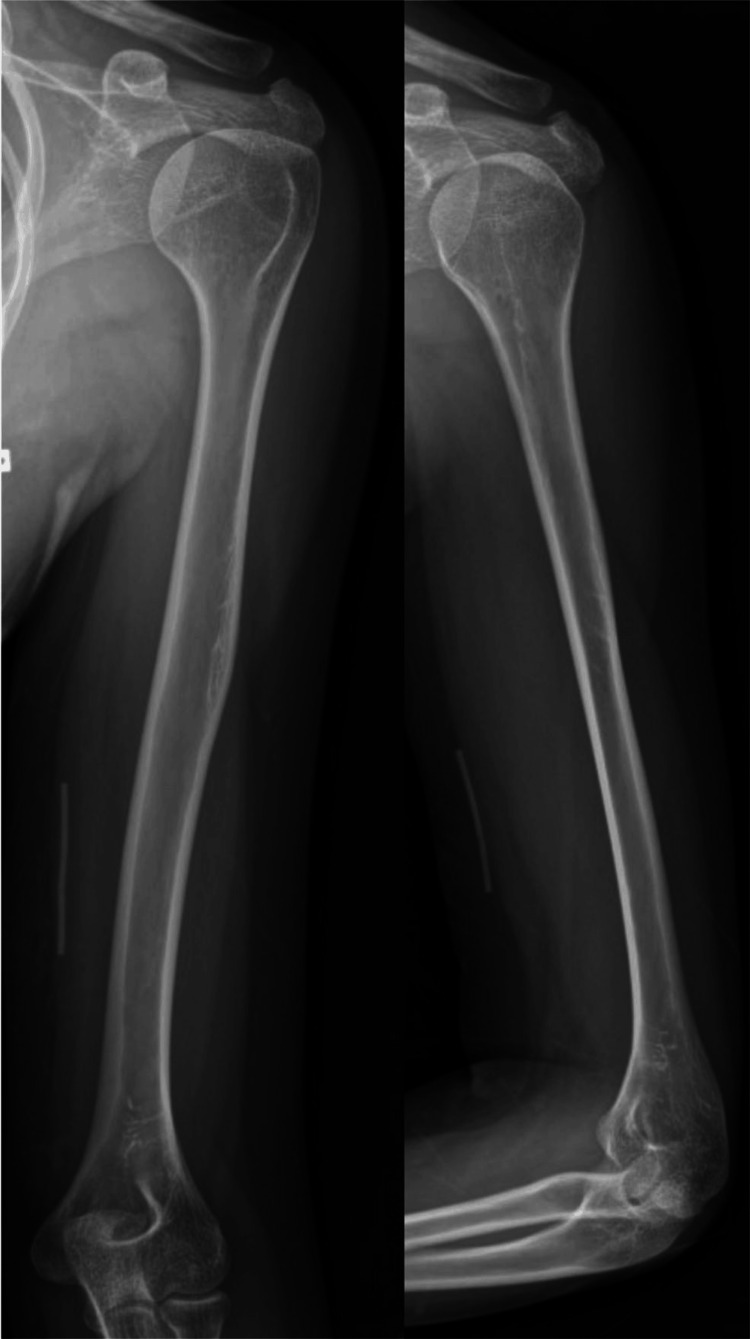
Radiograph of left humerus (anteroposterior and lateral views) showing the retained Implanon over the medial humerus

**Figure 2:**
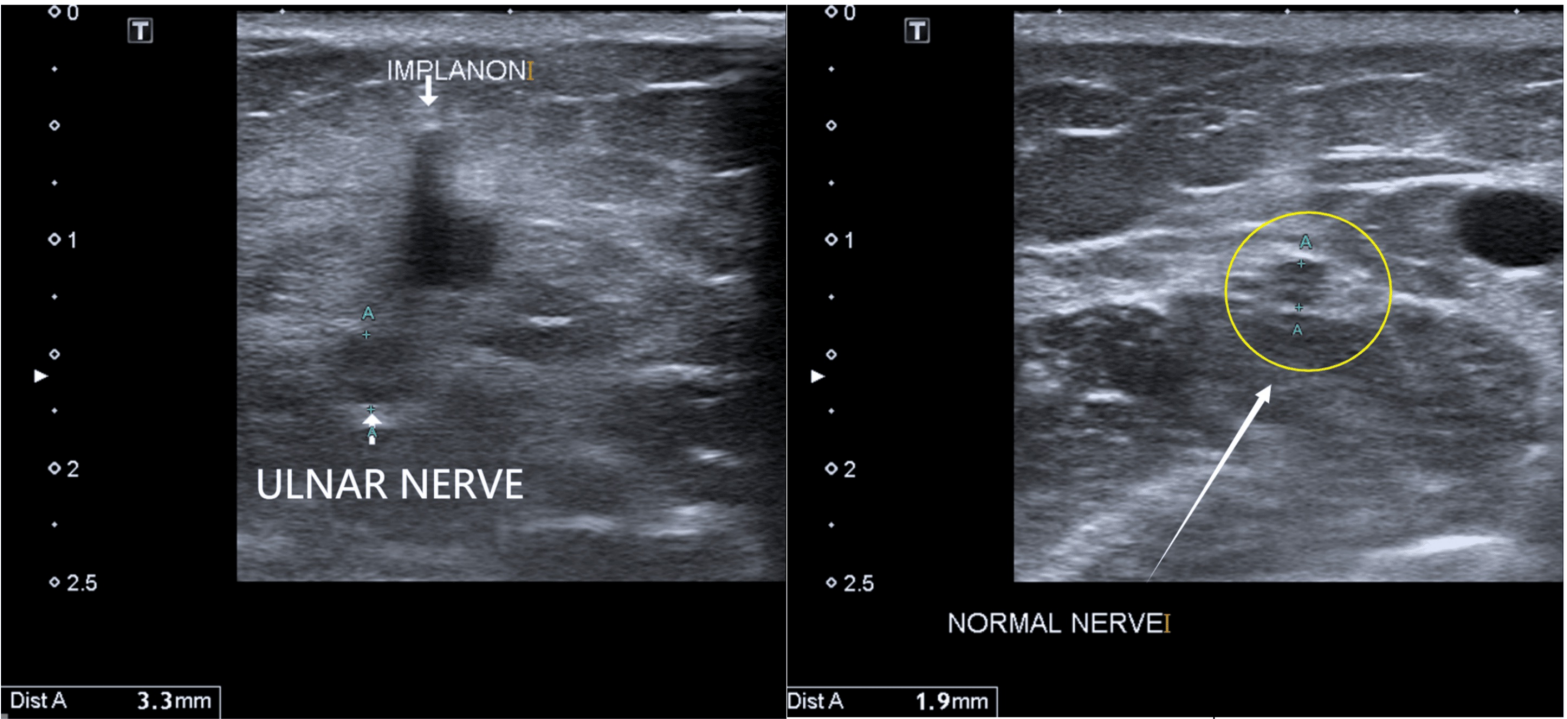
Ultrasound of the left arm showing the ulnar nerve measuring 3 mm compared to the normal 2 mm

Radiographs of the left arm showed a radio-opaque tubular foreign body over the distal medial humerus (Figure 1). Ultrasound imaging confirmed that the ulnar nerve was in continuity but appeared edematous, measuring 3 mm compared to the normal 2 mm (Figure 2).

**Figure 3:**
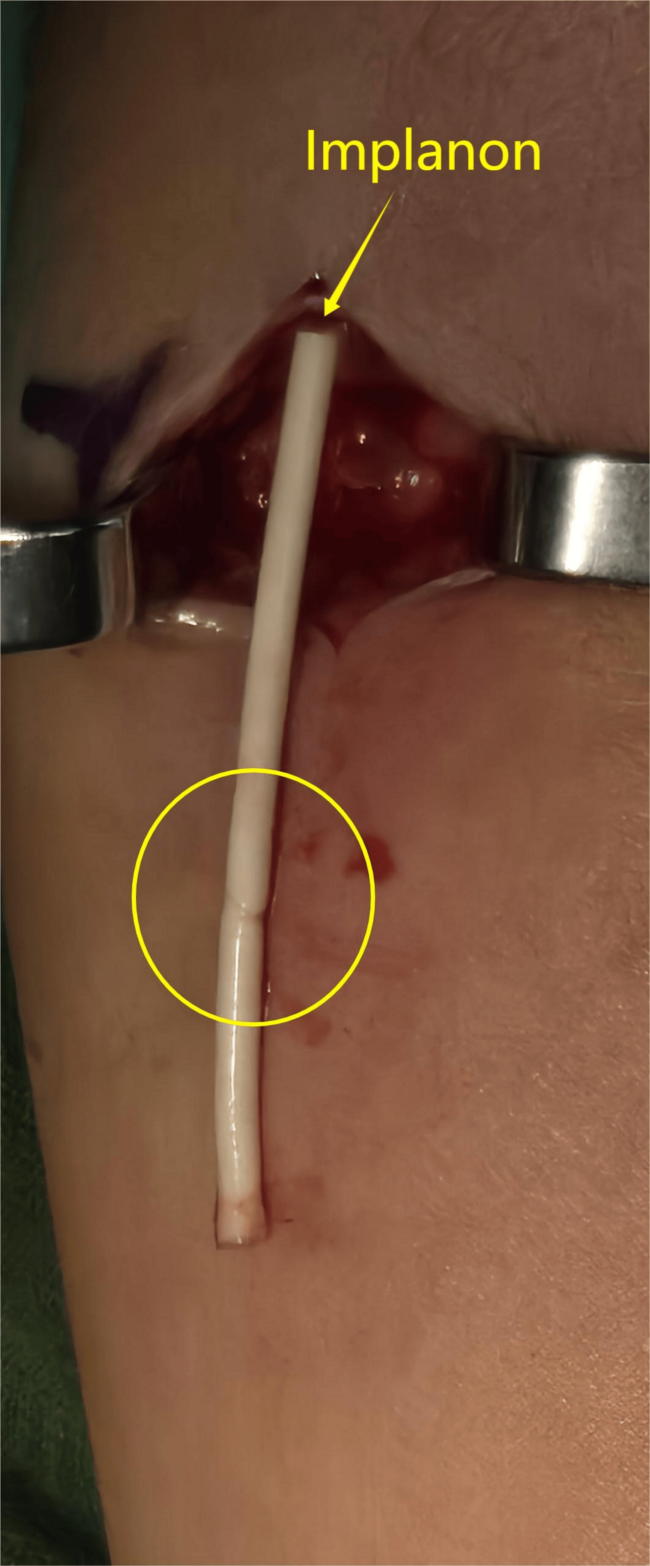
Implanon was removed through the previous scar over the medial aspect of the left arm The yellow circle highlights the partial transection mark on the Implanon

Under general anesthesia, the patient underwent surgical exploration of her left arm for removal of the Implanon device. A distal incision was made to directly visualize the implant tip. The device was located in the subcutaneous tissue at the original insertion site, where a partial transection from the previous procedure was observed (Figure 3).

**Figure 4:**
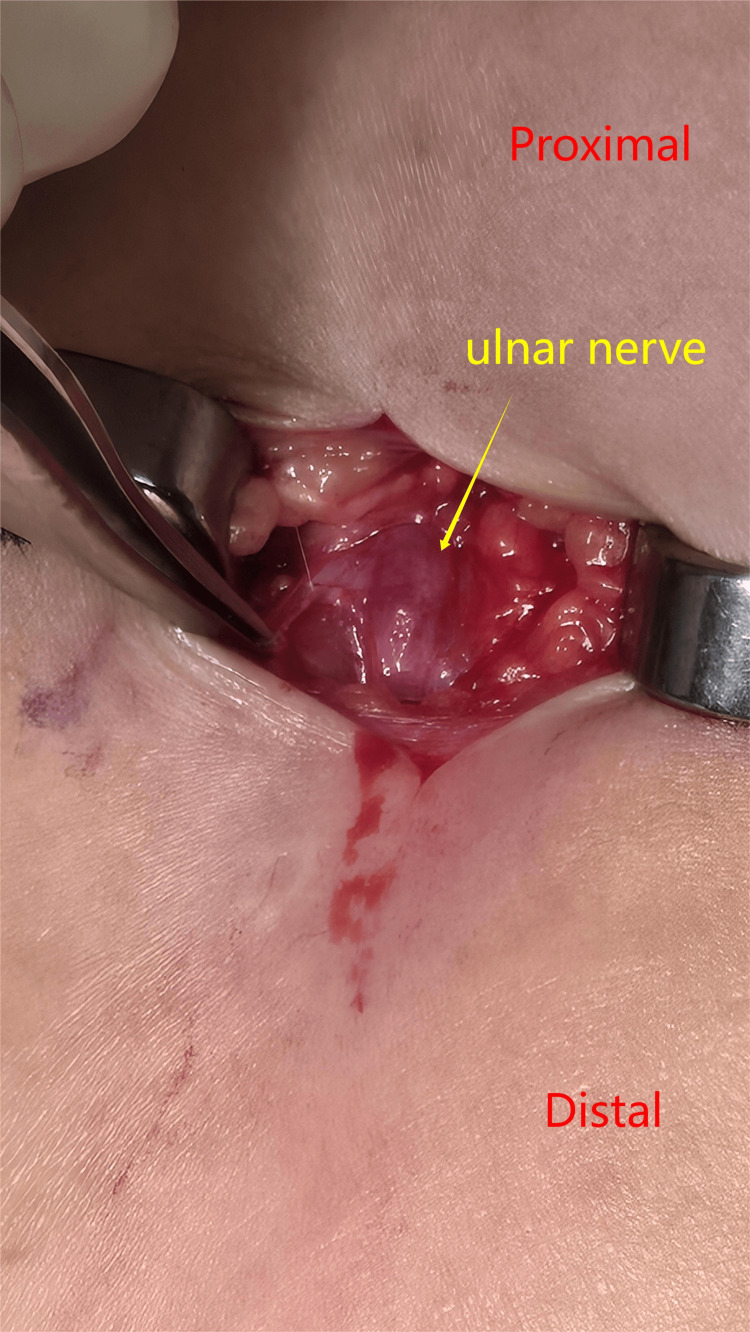
Ulnar nerve on exploration

The Implanon was successfully removed in one intact piece. Additionally, examination of the ulnar nerve revealed that it remained in continuity but exhibited signs of bruising and edema (Figure 4).

On the first postoperative day, the FDP strength improved to M5 in the ring finger and M4 in the little finger, although reduced sensation persisted over the little finger and the dorsal branch of the ulnar nerve. At the three-week follow-up, FDP strength remained unchanged, while the ADM muscles improved to M2. Sensory deficits in the dorsal branch of the ulnar nerve, however, continued to be evident.

## Discussion

Subdermal contraceptive implants, such as Implanon, are typically inserted in the subcutaneous plane along the medial aspect of the non-dominant arm, around 8-10 cm proximal to the medial epicondyle, under local anesthesia [1]. The implant should remain palpable throughout its use and is generally scheduled for removal after three years. The standard removal technique involves creating a small subcutaneous incision near the distal end of the implant, manually advancing the rod through the incision, and then grasping it with forceps [2].

Removal of non-palpable implants poses a greater challenge and should be performed only by physicians with experience in such procedures. Difficult removals can lead to complications including hematoma, local infections, and, in rare cases, nerve damage. Darney et al. [3] reported adverse events in less than 1% of insertions and 0.7% of removals.

In a study by Laumonerie et al. [4], nerve injuries were documented in 11 patients during removal and in one patient during insertion, with the medial antebrachial cutaneous and median nerves most affected. These injuries were primarily due to inadvertent traction or grasping of a nerve mistaken for the implant. In our case, we suspect that the ulnar nerve was injured during an attempted removal, as suggested by the partial transection of the implant.

Similarly, Hussain and Holland [5] described a case of severe ulnar nerve injury following an attempted extraction, which ultimately required an autologous sural nerve graft. Loss of ulnar nerve function can be devastating, given its essential role in both extrinsic and intrinsic hand muscle function and sensory perception.

Accurate localization of the implant is crucial before any extraction attempt. Physicians with appropriate training should supervise or perform the placement and removal of Implanon. Imaging studies should be employed to precisely locate non-palpable or migrated devices prior to removal, especially in patients who develop neurological symptoms. X-ray and ultrasound are both accessible and cost-effective, with ultrasound offering the added benefit of zero radiation exposure [1]. A study by Vidin et al. [6] found that from 28 operatively removed implants, 30% of them had migrated from the original insertion site, with 37% located intramuscularly while another 11% found within the neurovascular sheath.

It is also recommended that the exact location of the implant be documented via a thorough physical examination immediately after insertion and before extraction. If a patient presents with nerve symptoms in the context of a non-palpable or migrated implant, it is recommended to always use X-ray or ultrasound to determine the location before removal accurately. It is also strongly advised to refer early to a specialist center with a hand and microsurgery surgeon to assist in the removal of the migrated Implanon. This can help lower the risk of nerve injury and improve overall results.

## Conclusions

This case report highlights the development of ulnar nerve neuropathy following the removal of an Implanon device, underscoring the importance of recognizing potential complications associated with the procedure. Timely diagnosis and prompt, appropriate management are essential for improving patient outcomes and minimizing the risk of long-term neurological sequelae.
